# Characteristics of drug safety alerts issued by the Spanish Medicines Agency

**DOI:** 10.3389/fphar.2023.1090707

**Published:** 2023-01-30

**Authors:** Eva Montané, Javier Santesmases

**Affiliations:** ^1^ Department of Clinical Pharmacology, Hospital Universitari Germans Trias i Pujol, Barcelona, Spain; ^2^ Department of Pharmacology, Therapeutics and Toxicology, Universitat Autònoma de Barcelona, Barcelona, Spain; ^3^ Department of Internal Medicine, Hospital Universitari Germans Trias i Pujol, Barcelona, Spain; ^4^ Department of Medicine, Universitat Autònoma de Barcelona, Barcelona, Spain

**Keywords:** pharmacovigilance, safety-related regulatory actions, adverse drug reaction, spontaneous reporting systems, post-marketing surveillance

## Abstract

**Objectives:** To describe the characteristics of safety alerts issued by the Spanish Medicines Agency (AEMPS) and the Spanish Pharmacovigilance System over a 7-year period and the regulatory actions they generated.

**Methods:** A retrospective analysis was carried out of drug safety alerts published on the AEMPS website from 1 January 2013 to 31 December 2019. Alerts that were not drug-related or were addressed to patients rather than healthcare professionals were excluded.

**Results:** During the study period, 126 safety alerts were issued, 12 of which were excluded because they were not related to drugs or were addressed to patients and 22 others were excluded as they were duplications of previous alerts. The remaining 92 alerts reported 147 adverse drug reactions (ADRs) involving 84 drugs. The most frequent source of information triggering a safety alert was spontaneous reporting (32.6%). Four alerts (4.3%) specifically addressed health issues related to children. ADRs were considered serious in 85.9% of the alerts. The most frequent ADRs were hepatitis (seven alerts) and congenital malformations (five alerts), and the most frequent drug classes were antineoplastic and immunomodulating agents (23%). Regarding the drugs involved, 22 (26.2%) were “under additional monitoring.” Regulatory actions induced changes in the Summary of Product Characteristics in 44.6% of alerts, and in eight cases (8.7%), the alert led to withdrawal from the market of medicines with an unfavorable benefit/risk ratio.

**Conclusion:** This study provides an overview of drug safety alerts issued by the Spanish Medicines Agency over a 7-year period and highlights the contribution of spontaneous reporting of ADRs and the need to assess safety throughout the lifecycle of medicines.

## 1 Introduction

Drug safety is an important health concern that requires continuous evaluation throughout a drug’s lifecycle, not only during its development phase but also after it has entered the market. When a drug is approved, safety information is still limited. A major reason for this is the poor external validity of clinical trials, particularly for some conducted by the pharmaceutical industry (Rothwell, 2005). In these studies, patients exposed to the drug have been selected following strict inclusion and exclusion criteria, leading to significant differences between the population treated and the individuals likely to be seen in real clinical practice who tend to be older, have many comorbidities, and use polypharmacy (Martin et al., 2004). Some adverse drug reactions (ADRs) may only be detected after marketing authorization has been granted because of the mechanism of production, a long latency period, or very low incidence. Therefore, the safety of medicines should be continuously assessed even after their authorization and appearance on the market, through pharmacovigilance systems (European Medicines Agency, 2022a) in which governmental regulatory agencies typically play a key role. In the last 50 years, pharmacovigilance systems have been developed in many countries around the world (Uppsala Monitoring Centre, 2022). In the European Union (EU), pharmacovigilance is jointly managed by EU member states, the European Medicines Agency (EMA), and the European Commission (European Medicines Agency, 2022a).

When health professionals or patients suspect an ADR, a spontaneous report is sent to the local regulatory agency. In Spain, reports are first sent to the national pharmacovigilance system the *Sistema Español de Farmacovigilancia de Medicamentos de uso Humano* (SEFV-H). The SEFV-H consists of 17 regional pharmacovigilance centers and the Spanish Medicine Agency (*Agencia Española de Medicamentos y Productos Sanitarios* [AEMPS]). Then, reports are sent from the SEFV-H to EudraVigilance, the EMA’s pharmacovigilance network. Finally, spontaneous reports are included in Vigibase, the database of the World Health Organization Uppsala Monitoring Centre (Baldo et al., 2018).

To ensure that drugs continue to have an optimal benefit–risk balance after they are put on the market, they are typically subject to risk management plans involving post-authorization safety studies. However, spontaneous reports of ADRs are also essential in triggering alarm signals. Then, these signals need to be detected, validated, and confirmed following a management process (European Medicines Agency, 2022b). For this purpose, the EMA published new legislation concerning pharmacovigilance and risk management in 2010 and in the process created the Pharmacovigilance Risk Assessment Committee (PRAC), which is the EU-level committee responsible for assessing and monitoring the safety of medicinal products for human use across Europe. The PRAC came into existence at the end of 2012 and has since demonstrated its effectiveness in detecting and evaluating new drug safety signals (Potts et al., 2020). In Spain, the published law was enacted in 2013 that brought Spain in line with European legislation (Agencia estatal boletín oficial del estado, 2022) by promoting the creation of health data bases, conducting pharmacoepidemiological studies and evaluating ADR reports received by the SEFV-H.

In general, drug safety alerts are motivated by signals obtained from databases of pharmacovigilance systems and signals warning of ADRs detected in post-marketing studies and/or clinical trials. A signal “suggests a new potentially causal association or a new aspect of a known association between an intervention and an event or set of related events that is judged to be of sufficient likelihood to justify verificatory action” (European Medicines Agency, 2022b). The generated alerts provide information to health-care professionals or patients about the needed regulatory actions based on the signals such as new restrictions on the use of medicine in particular populations, the mandatory monitoring of patients using that medicine, or the withdrawal of the drug from the market (Farcaş et al., 2018).

In a recent study comparing drug safety alerts issued by national medicine regulators in Australia, Canada, the United Kingdom, and the United States from 2007 to 2016, major differences were found in the use of safety advisories by regulators, including their frequency, content, communication type, and focus (Perry et al., 2020). However, few previous studies have analyzed the characteristics of safety alerts issued by regulatory authorities, though data are available for a few other countries, including Portugal, the Netherlands, Brazil, and the Ivory Coast (Ferreira et al., 2020; Soares et al., 2015; Mol et al., 2010; N’Guessan-Irié et al., 2012). To our knowledge, no research has been published regarding alerts specifically issued by the Spanish Medicines Authority.

With the goal of filling the gap, this study attempted to describe the characteristics of safety warnings issued by the Spanish Medicines Agency and Pharmacovigilance System during a 7-year period after PRAC began to function and examine the regulatory actions taken as a result of each alert. Secondary goals included comparing the alerts issued by different institutions, assessing specific safety alerts related to drugs under ‘additional monitoring,’ and analyzing alerts according to the drug’s target population and medical area of focus.

## 2 Methods

### 2.1 Design

This study is a retrospective analysis of drug safety alerts issued by the Spanish Medicines Agency and published on its website (Notas informativas and medicamentos de uso humano. Agencia Española de Medicamentos y Productos Sanitarios, 2022) during the 7-year period from 1 January 2013, after the launch of the PRAC to 31 December 2019, and before the COVID-19 pandemic started. This cut-off date was chosen to include homogenous safety alerts.

All of the safety alerts issued were selected and those involving medicines were included. Alerts that were not drug-related, warnings addressed to patients, and duplicated alerts were excluded. We considered an alert was duplicated when the ADR alert was similar and related to the same drug.

### 2.2 Variables

For each alert, the following descriptive variables were recorded.

- Year of publication
- Issuing agency: AEMPS or EMA
- Population targeted by the alert: children <18 years old, adults 18–65 years old, and elderly >65 years old
- Drugs involved: classification was based on the first level of the Anatomical Therapeutic Chemical (ATC) classification (WHO Collaborating Centre for Drug Statistics Methodology, 2018)
- Limitations on the prescription or dispensing of the drug: specifically whether it was limited to the hospital setting or not
- Prescribing specialists: the healthcare professionals most likely to prescribe the drug in question in the consensus view of the authors; for widely used drugs, prescribing professionals were assumed to be general practitioners
- Drugs under “additional monitoring:” this term is used by the EMA to denote medicines that are more intensively monitored than others, generally because there is less safety information available; they have an inverted black triangle [▼] displayed on their packaging and Summary of Product Characteristics (SmPC) (European Medicines Agency, 2022)
- Drug indication: A medical condition for which that medicine is prescribed
- Type of ADR: classified in accordance with the System Organ Classes (SOC) of the Medical Dictionary for Regulatory Activities (MedDRA) (MEDDRA, 2021)
- Seriousness of the ADR: a serious ADR was defined according to the International Conference on Harmonization (ICH) guideline E2D as one that is fatal, life-threatening, requires hospital admission or prolongation of hospital stay and causes persistent or significant disability/incapacity, congenital anomaly/congenital defect or medically important (European Medicines Agency, 2004)
- Sources of evidence: spontaneous reports, clinical trials, and/or observational studies
- Reiteration of the alert: whether it was a first-time alert or the reiteration of an alert issued before the study period
- Regulatory actions to be taken as noted in the alert such as new restrictions on use, risk minimization measures, changes in SmPC, or withdrawal from the market. Regulatory actions are based on the scientific decisions of an expert group responsible for assessing safety problems related to medicines such as the Committee for Medicinal Products for Human Use [CHMP]. When the group decides that the degree of risk is acceptable under the currently authorized conditions of use, the regulatory action is to provide information regarding the ADR and recommending risk minimization measures such as patient monitoring and follow-up. When the group decides that the risk is acceptable but only under certain conditions, the regulatory agency issues a restriction of use such as restricting the indications for which the medicine may be prescribed or the patient population to which it may be given. Any of these measures can lead to the modification and/or updating of the SmPC. When the group decides that the risk is unacceptable and the benefit/risk ratio is unfavorable, the drug is withdrawn from the market (Rodriguez Pascual, 2006).

The selection of alerts for inclusion in the study and the recording of study variables were conducted by the two researchers working independently (SJ and ME). Consensus was reached whenever discrepancies arose.

### 2.3 Statistical analysis

Descriptive statistics (counts and percentages) were used to characterize the variables assessed in this study. To compare the characteristics of alerts issued by AEMPS with those issued by the EMA (mainly by the PRAC), the Pearson chi-square or Fisher’s exact test for categorical variables was used. A bilateral *p*-value <0.05 was used to determine statistical significance.

All statistics were performed using the SPSS software package for Windows version 19.0 (SPSS Inc., Chicago, Ill).

## 3 Results

**FIGURE 1:**
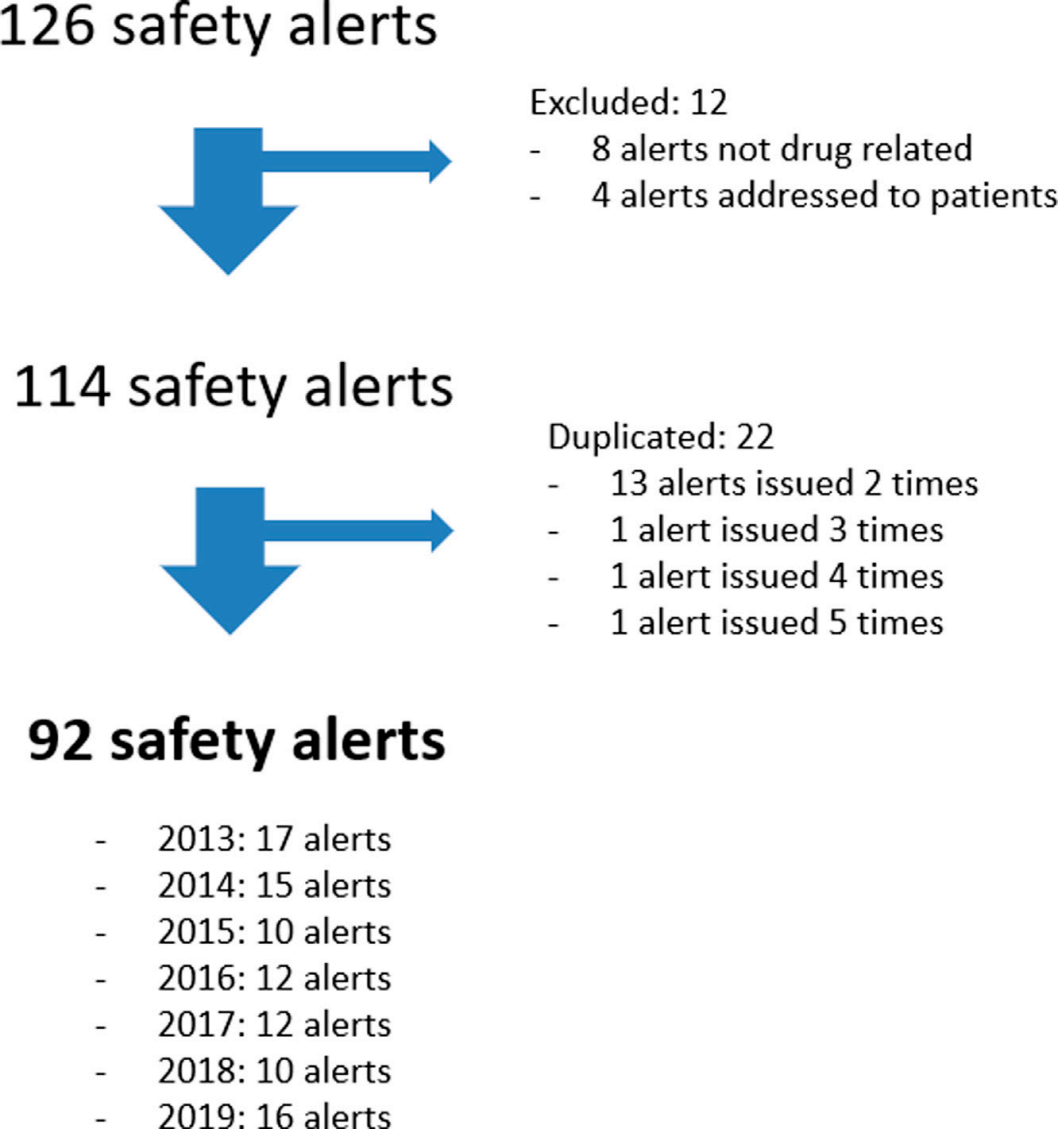
Flow chart of included safety alerts.

An initial search of the EMA website yielded a total of 126 safety alerts. Thirty-four alerts were excluded (22 were duplicated alerts) (Figure 1). These duplicated alerts provide new safety data, corroborate drug risks reported by the PRAC when issued by the AEMPS, or include changes to the regulatory measures applied. Drugs involved in duplicated alerts were alemtuzumab, canagliflozin, cilostazol, codeine, cyproterone acetate, denosumab, diacerein, fentanyl, fingolimod, fusafungine, gliflozins, hydroxyethylstarch, idelalisib, methotrexate, mycophenolic acid, radium dichloride, strontium ranelate, tetrazepam, tofacitinib, ulipristal, valproic acid, and VPH vaccine. All duplicates (*n* = 22) were eliminated from the analyzed dataset, which resulted in a total of 92 remaining alerts involving 84 drugs.

Forty alerts (43.5%) were issued by the AEMPS, while the remainder were issued by the EMA (47 of which were specifically issued by the PRAC). Sources used to support safety alerts were spontaneous reporting of ADRs (30 alerts, 32.6%), clinical trials (16 alerts, 17.4%), observational studies (eight alerts, 8.7%), or multiple sources (21, 22.8%). A total of 17 alert (18.5%) sources were not specified.

A total of 147 ADRs were reported in the 92 safety alerts, ranging from one to four ADRs per alert; two or more ADRs were reported in 42 (45.6%) alerts. The most frequently reported ADRs were hepatitis (nine alerts), death (seven alerts), congenital malformation (six alerts), and drug inefficacy, arrhythmia, and arterial thrombosis (five alerts each). The remaining types of ARDs are detailed in Table1. The most frequent ADRs in terms of the MedDRA’s SOC classification were cardiac, vascular, and hepatobiliary disorders (19, 15, and 14 alerts, respectively) (Table 2).

**TABLE 1:** Type of adverse drug reaction (ADR) related to the alert.

ADR	Number of alerts	% ADR
Hepatitis	9	6.1
Death	7	4.8
Congenital malformation	6	4.1
Inefficacy	5	3.4
Arrhythmia	5	3.4
Arterial thrombosis	5	3.4
Allergic reaction	4	2.7
Cardiovascular risk	4	2.7
Progressive multifocal leukoencephalopathy	4	2.7
Venous thrombosis	4	2.7
Acute myocardial infarction	3	2.1
Cardiac failure	3	2.1
Cerebral deposit	3	2.1
Drug toxicity	3	2.1
Infection	3	2.1
Bleeding	2	1.4
Bone fracture	2	1.4
Hypotension	2	1.4
Kidney failure	2	1.4
Lymphoma	2	1.4
Liver failure	2	1.4
Mandibular osteonecrosis	2	1.4
Poisoning	2	1.4
Psychiatric disorder	2	1.4
Skin cancer	2	1.4
Skin reaction	2	1.4
Abuse and dependence, acute pulmonary edema, agranulocytosis, alveolar bleeding, atrial fibrillation, atrioventricular block, bradycardia, bronchiectasis, cirrhosis, coma, complex regional pain syndrome (CRPS), connective tissue fibrosis, convulsions, cutaneous reaction, diabetes mellitus, diarrhea, difficulty driving, ergotism, extrapyramidalism, hepatocarcinoma, hemophilia, hypercalcemia, hyperkalemia, hypersensitivity, hypertrichosis, hypocalcemia, hypogammaglobulinemia, hyponatremia, ketoacidosis, liver decompensation, liver toxicity, lower extremity amputation, lung disease, lymphohistiocytosis, miscarriage, muscle weakness, nephrotic syndrome, neural tube defects, neurodevelopmental disorder, ocular pigmentation, opportunist infection, pancreatitis, Parkinson’s disease, pneumonia, pneumonitis, postural orthostatic tachycardia syndrome (PoTS), pulmonary fibrosis, pulmonary infection, pulmonary thromboembolism, respiratory depression, restless legs, skin pigmentation, somnambulism, stroke, tendinopathy, thrombocytopenia, thrombotic microangiopathy, tumor, VHB reactivation	1 each (56)	0.68% each
Total	147	100

**TABLE 2:** ADRs classified according to MedDRA’s SOC terminology.

	Number of alerts	%
**Infections and infestations**	10	7
Infections (3), progressive multifocal leukoencephalopathy (4), opportunistic infection (1), pulmonary infection (1), and VHB reactivation (1)		
**Neoplasms benign, malignant, and unspecified**	7	4.9
Lymphoma (2), skin cancer (2), hepatocarcinoma (1), lymphohistiocytosis (1), and tumor (1)		
**Blood and lymphatic system disorders**	6	4.2
Hemorrhage (2), agranulocytosis (1), hemophilia (1), thrombocytopenia (1), and thrombotic microangiopathy (1)		
I**mmune system disorders**	6	4.2
Allergic reaction (4), hypersensitivity (1), and hypogammaglobulinemia (1)		
**Endocrine disorders**	4	2.8
Hypercalcemia (1), hypocalcemia (1), hyponatremia (1), and hyperkaliemia (1)		
**Metabolism and nutrition disorders**	2	1.5
Diabetes mellitus (1) and ketoacidosis (1)		
**Psychiatric disorders**	4	2.8
Abuse and dependence (1), psychiatric disorder (2), and somnambulism (1)		
**Nervous system disorders**	9	6.3
Cerebral deposit (3), coma (1), convulsions (1), extrapyramidal symptoms (1), neurodevelopmental disorder (1), Parkinson’s (1), and restless legs (1)		
**Eye disorders**	1	0.7
Ocular pigmentation (1)		
**Ear and labyrinth disorders**	0	0
**Cardiac disorders**	19	13.4
Arrhythmia (5), cardiovascular risk (4), acute myocardial infarction (3), cardiac failure (3), acute pulmonary edema (1), atrial fibrillation (1), atrioventricular block (1), and bradycardia (1)		
**Vascular disorders**	15	10.6
Arterial thrombosis (5), venous thrombosis (4), hypotension (2), ergotism (1), lower extremity amputation (1), postural tachycardia syndrome (PoTS) (1), and pulmonary thromboembolism (1)		
**Respiratory, thoracic, and mediastinal disorders**	7	4.9
Alveolar bleeding (1), bronchiectasis (1), pneumonia (1), pneumonitis (1), lung disease (1), pulmonary fibrosis (1), and respiratory depression (1)		
**Gastrointestinal disorders**	2	1.5
Diarrhea (1) and pancreatitis (1)		
**Hepatobiliary disorders**	14	9.9
Hepatitis (9), liver failure (2), hepatic cirrhosis (1), hepatic decompensation (1), and liver toxicity (1)		
**Skin and subcutaneous tissue disorders**	4	2.8
Skin reaction (2), hypertrichosis (1), and skin pigmentation (1)		
**Musculoskeletal and connective tissue disorders**	8	5.6
Bone fracture (2), mandibular osteonecrosis (2), complex regional pain syndrome (CRPS) (1), connective tissue fibrosis (1), muscle weakness (1), and tendinopathy (1)		
**Renal and urinary disorders**	3	2.2
Kidney failure (2) and nephrotic syndrome (1)		
**Pregnancy, puerperium, and perinatal conditions**	1	0.7
Miscarriage (1)		
**Reproductive system and breast disorders**	0	0
**Congenital, familial, and genetic disorders**	7	4.9
Congenital malformation (6) and neural tube defect (1)		
**General disorders and administration site conditions**	7	4.9
Death (7)		
**Investigations**	0	0
**Injury, poisoning, and procedural complications**	5	3.5
Drug toxicity (3) and poisoning (2)		
**Surgical and medical procedures**	0	0
**Social circumstances**	1	0.7
Difficulty driving (1)		
**Product issues**	0	0
	142^a^	100

^a^ 5 alerts were not classifiable (alerts of drug inefficacy).

The most frequently alerted drugs (by ATC category) were L-Antineoplastic and immunomodulating agents (21 alerts, 23%), N-Nervous system (12, 13%), and J-Anti-infectives for systemic use (11, 12%) (Table 3). The most alerts (three) were issued for fingolimod (Table 4). The prescription of 37 drugs (45%) was restricted to the hospital setting.

**TABLE 3:** Anatomical Therapeutic Chemical (ATC) drug classification system.

ATC category	Therapeutic area	Number of alerts	%
A	Alimentary tract and metabolism	7	7.6
B	Blood and blood forming organs	9	9.8
C	Cardiovascular system	6	6.5
D	Dermatological	1	1.1
G	Genito-urinary system and sex hormones	7	7.6
H	Systemic hormonal preparations, excluding sex hormones and insulin	3	3.3
J	Anti-infectives for systemic use	11	12
L	Antineoplastic and immunomodulating agents	21	22.8
M	Musculoskeletal system	9	9.8
N	Nervous system	12	13
P	Antiparasitic products, insecticides, and repellents	0	0
R	Respiratory system	2	2.2
S	Sensory organs	0	0
V	Various	4	4.3
	Total	92	100

**TABLE 4:** Drugs involved in alerts.

Drug	ATC code	Number of alerts	%
Fingolimod	L04AA27	3	3.2
Cilostazol	B01AC23	2	2.1
Codeine	R05DA04	2	2.1
Denosumab	M05BX04	2	2.1
Fentanyl	N02AB03	2	2.1
Mycophenolic acid	L04AA06	2	2.1
Tofacitinib	L04AA29	2	2.1
Parenteral iron	B03AC	2	2.1
Progestogens and estrogens, fixed combinations	G03AA	2	2.1
Aceclofenac, aliskiren/ACE inhibitors/sartans, aflibercept, agomelatine, alemtuzumab, atosiban, brivudine, bromocriptine, calcitonin, canagliflozin, carbimazole/thiamazole, cladribine, cholecalciferol/calcidiol, coagulation factor VIII, corticosteroids, cyproterone acetate and estrogen, daratumumab, diacerein, diclofenac, dimethyl fumarate, direct acting antivirals, direct factor Xa inhibitors, dolutegravir, domperidone, elvitegravir/cobicistat, ergot alkaloids, febuxostat, flutamide, fusafungine, gadobenic acid, gadodiamide, gadolinium, gliflozins, hydrochlorothiazide, hydroxyzine, hydroxyethylstarch, ibuprofen/dexibuprofen, idelalisib, interferon beta, immunosuppressants, Inzitan®, ivabradine, ketoconazole, leuprorelin, metamizole, methylprednisolone, metoclopramide, methotrexate, natalizumab, nicotinic acid-laropiprant, nitrofurantoin, ombitasvir/paritaprevir/ritonavir, omeprazole, ondansetron, papillomavirus vaccine, parenteral nutrition, pomalidomide, posaconazole, quinolone, radium dichloride, retigabine, retinoids, riociguat, ritodrine, selexipag, sofosbuvir/ledipasvir/daclatasvir–amiodarone, strontium ranelate, tetrazepam, trimetazidine, tyrosine kinase inhibitors, ulipristal, valproic acid, and zolpidem	B01AC06	1 each (73)	1.1% each
Total	-	92	100

Inzitan®: dexamethasone, thiamine, cyanocobalamin, and lidocaine

With regard to drugs under ‘additional monitoring,’ a total of 26 alerts (28.3%) were related to 22 of these drugs (Table 5), and the most frequently involved drugs were fingolimod (three alerts) and tofacitinib (two alerts). Hepatitis or hepatobiliary disorders were the most frequent ADR alerts for this group of drugs (in connection with five drugs, 22.7%). The SmPC was modified for 12 of these drugs (54.5%), and one of them (strontium ranelate) was withdrawn from the market.

**TABLE 5:** Characteristics of alerts for drugs under ‘additional monitoring.’

ADR	Drug	ATC first level	Modification of the SmPC
Osteonecrosis mandibular	Aflibercept	L	Yes
Hepatitis and hepatocarcinoma	Direct agent antiviral	J	No
Ketoacidosis	Canagliflozin and other gliflozins	A	Yes
Limb amputation			No
Cardiovascular risk and hemorrhage	Cilostazol	C	Yes
Hepatitis	Daratumumab	L	Yes
Osteonecrosis and hypocalcemia	Denosumab	M	No
Neural tube defect	Dolutegravir	J	No
Arrhythmia, lymphoma, congenital malformation, skin cancer, and opportunistic infection	Fingolimod	L	Yes
Infection	Idelalisib	L	No
VHB reactivation	Immunosuppressants	L	No
Hypersensitivity	Iron isomalthose	B	No
Progressive multifocal leukoencephalopathy	Natalizumab	L	Yes
Liver failure and liver decompensation	Paritaprevir/ombitasvir/ritonavir	J	Yes
Hepatitis	Pomalidomide	L	Yes
Bone fracture and death	Radium dichloride	V	No
Ocular and skin pigmentation	Retigabine	N	No
Death and respiratory infection	Riociguat	C	Yes
Toxicity and inefficacy	Selexipag	L	No
Bradycardia and cardiac block	Sofosbuvir/ledipasvir/daclatasvir– amiodarone	J	Yes
Cardiovascular risk and skin reaction	Strontium ranelate	M	Market withdrawal
Pulmonary thromboembolism and death	Tofacitinib	L	Yes
Venous thrombosis and infection			No
Hepatitis	Tyrosine kinase inhibitors	L	Yes

SmPC, summary of product characteristics

All ADRs were considered serious, with the six exceptions being drug inefficacy, cerebral deposits of contrast, and hypertrichosis. Thirty-nine (42.4%) alerts were addressed to adult populations, 37 (40.2%) were addressed to the elderly population (whether exclusively or not), four (4.3%) alerts were specifically addressed to children or to children and adults, and eight (8.7%) alerts were targeted at populations of all ages. Alerts addressed to children are detailed in Table 6. Seven (7.6%) alerts were addressed to pregnant women and warned of consequences to the fetus (mainly congenital malformations). The features of these alerts are detailed in Table 7.

**TABLE 6:** Characteristics of alerts with drugs addressed to children.

Year of issue	Drug	ADR	Mechanism of ADR	Issuing institution	Source	Regulatory action
2013	Codeine (analgesic)	Respiratory depression death	Overdose due to genetic deficiency of CYP2D6	PRAC	Spontaneous reporting and others	Restriction by age and diseases
2015	Codeine (antitussive)	Intoxication	Overdose due to genetic deficiency of CYP2D6	PRAC	Spontaneous reporting	Restrictions in use for ages <12
2019	Parenteral nutrition	Death	Peroxide formation due to exposure to light	AEMPS	Laboratory and clinical studies	Changes in the SmPC
2019	Omeprazole	Hypertrichosis	There was minoxidil instead of omeprazole due to a manufacturing error	AEMPS	Spontaneous reporting	Withdrawal of batches

AEMPS, *Agencia Española de Medicamentos y Productos Sanitarios*; PRAC, pharmacovigilance risk assessment committee; SmPC, summary of product characteristics

**TABLE 7:** Characteristics of alerts with drugs addressed to pregnant women.

Year of issue	Drug	ADR	Issuing institution	Source of information	Regulatory action
2014 and 2018	Valproic acid	Congenital malformation	PRAC	Observational studies	Use restrictions, risk minimization measures
		Neurodevelopmental disorder			
2015	Mycophenolate mofetil	Congenital malformation	AEMPS	Not specified	Use restrictions, risk minimization measures
		Miscarriage			
2018	Retinoids	Congenital malformation	PRAC	Not specified	Use restrictions, risk minimization measures
2019	Elvitegravir + cobicistat	Inefficacy	AEMPS	Clinical trial	Use restrictions
		Risk of mother-to-child transmission of HIV			
2019	Fingolimod	Congenital malformation	PRAC	Not specified	Use restrictions
2019	Ondansetron	Congenital malformation	PRAC	Observational studies	Use restrictions
2019	Carbimazol/tiamazol	Congenital malformation	AEMPS	Observational studies and spontaneous reporting	Use restrictions, risk minimization measures

AEMPS, *Agencia Española de Medicamentos y Productos Sanitarios*; HIV, human immunodeficiency virus; PRAC, pharmacovigilance risk assessment committee

The prescribing specialists were most often rheumatologists (10 alerts), neurologists, and hematologists (eight alerts each), although 11 alerts involved drugs to treat unspecific symptoms such as pain or fever, emesis, cough, rhinosinusitis, and urinary tract infection and therefore were likely of interest to general practitioners. The diseases most likely linked to safety alerts were any kind of pain (seven alerts), multiple sclerosis (six alerts), and osteoporosis (five alerts) (Table 8).

**TABLE 8:** Indication of drugs involved in alerts and potential specialist prescriber.

Specialist prescriber	Indication (number of alerts)	Number of alerts	%
General practitioner	Pain or fever (5), emesis (2), cough (1), dyslipidemia (1), rhinosinusitis (1), and urinary tract infection (1)	11	11.9
Rheumatology	Osteoporosis (5), arthritis (3), hyperuricemia (1), and osteoarthritis (1)	10	10.8
Hematology	Leukemia (3), iron deficiency (2), multiple myeloma (2), and hemophilia (1)	8	8.6
Neurology	Multiple sclerosis (6)and epilepsy (2)	8	8.6
Obstetrics and gynecology	Contraception (2), preterm labor (2), lactation (1), uterine myoma (1), and prophylaxis HPV infection (1)	7	7.6
Infectious diseases	HIV infection (2), mycosis (2), and bacterial infection (1)	5	5.4
Cardiology	Angina (2), anticoagulation (1), cardiac failure (1), and diuretic (1)	5	5.4
Oncology	Immunosuppression (2), chemotherapy-induced emesis (1), colorectal cancer (1), and disruptive pain (1)	5	5.4
Dermatology	Acne and hirsutism (1), acne and psoriasis (1), and herpes zoster infection (1)	3	3.3
Endocrinology	Diabetes mellitus (2) and hyperthyroidism (1)	3	3.3
Pneumology	Pulmonary hypertension (2) and asthma/COPD (1)	3	3.3
Hepatology	VHC infection (3)	3	3.3
Pediatrics	Gastroesophageal reflux (1), malnutrition (1), and pain (1)	3	3.3
Psychiatry	Bipolar disorder (1), depression (1), and insomnia (1)	3	3.3
Radiology	NMR contrast (3)	3	3.3
Urology	Prostate cancer (3)	3	3.3
Allergology	Allergy (2)	2	2.2
Gastroenterology	Ulcerative colitis (2)	2	2.2
Vascular surgery	Intermittent claudication (2)	2	2.2
Intensive care medicine	Acute hemorrhage (1)	1	1.1
Nephrology	Kidney transplantation (1)	1	1.1
Traumatology	Musculoarticular pain (1)	1	1.1
Total		92	100

COPD, chronic obstructive pulmonary disease; HIV, human immunodeficiency virus; HPV, human papillomavirus; NMR, nuclear magnetic resonance; VHC, Hepatitis C virus.

The most frequent regulatory actions derived from alerts were “restrictions in use” in 30 alerts (32.6%) and “reporting of ADRs and recommendation of patient monitoring and follow-up” in 14 alerts (15.2%). ‘Restrictions in use’ included restrictions related to the age of the population receiving the medicine, the specific disease being treated, and the duration of treatment and/or dosage. Combinations of these regulatory actions were present in 22 alerts (23.9%). The regulatory actions of 41 alerts (44.6%) involved changes in the SmPC, and eight (8.7%) required mandated withdrawal from the market of medicines with an unfavorable benefit/risk ratio. Only one of these withdrawn medicines was put on the market in the 5 years prior to the alert, while five had been on the Spanish market for more than 20 years (Table 9). Two batches of one drug were withdrawn from the market because they contained minoxidil instead of omeprazole due to an error in the manufacturing process.

**TABLE 9:** Characteristics of medicines withdrawn from the market.

Year medicine came to the market	Year alert was issued	Issuing institution	Drug (medicine)	Indication	ADRs
2008	2013	PRAC	Nicotinic acid-laropiprant (Tredaptive®)▼	Hyperlipidemia	Highest incidence of bleeding, muscle weakness, infections, and diabetes mellitus
1978	2013	PRAC	Tetrazepam (Myolastan®)	Muscle pain	Cutaneous reaction
<1991	2013	PRAC	Ketoconazole systemic use	Mycosis	Hepatitis
1964	2016	PRAC	Fusafungine (Fusaloyos®)	Rhinopharyngitis	Allergic reaction
2004	2014	PRAC	Strontium ranelate (Protelos®) (Osseor®)	Osteoporosis	Cardiovascular risk and cutaneous reaction
1968	2017	AEMPS	Inzitan® (dexamethasone, thiamine, lidocaine, and cyanocobalamin)	Pain	Allergic reaction
1994	2018	PRAC	Gadodiamide (Omniscan®)	Magnetic resonance	Cerebral deposit
2019	2019	AEMPS	Batches with minoxidil instead of omeprazole (Farma-Química Sur S.L)	Gastroesophageal reflux	Hypertrichosis

AEMPS, *Agencia Española de Medicamentos y Productos Sanitarios*; PRAC, pharmacovigilance risk assessment committee

### 3.1 Comparison of alerts issued by the AEMPS with those issued by the EMA

When safety alerts were compared by the issuing institution, some significant differences were found. Alerts issued by the AEMPS involved more drugs ‘under additional monitoring’ (42.5% vs. 17.3%; *p* < 0.008) and drugs restricted to a hospital setting (60% vs. 34.6%; *p* < 0.015), while alerts issued by the EMA were more often addressed to child populations (25% vs. 7.5%; *p* < 0.028).

## 4 Discussion

The results of this study show that the Spanish Medicines Agency issued, on average, one or two drug safety alerts every month from 2013 to 2019, one out of five of these were alert duplications. The rate of alert issuance did not increase over time in this period, contrary to other studies conducted in Portugal and the Netherlands (Soares et al., 2015; Ferreira et al., 2020). Almost half of the alerts were issued by the national medicines agency, and some were subsequently issued by the EMA, indicating that the SEFV-H is a proactive local safety system and an effective national signal detection system that anticipates alerts issued by the PRAC (Farcas et al., 2020). Therefore, our findings are in line with those of other similar European studies. However, differences between European and other regulatory agencies (such as those in the US, Canada, or Australia) may lead to different risk assessments of a drug and hence to different regulatory actions (Farcaş et al., 2018; Bjerre et al., 2018; Bhasale et al., 2021).

In this study, we found that the main trigger leading to the generation of these alerts was spontaneous case reports, as indicated in other studies (Alves et al., 2013; Lester et al., 2013; Ishiguro et al., 2017). This underlines the importance and usefulness of spontaneous reporting and national pharmacovigilance systems in detecting issues of concern related to medicines and contributing to the generation of safety alerts during the marketing period (European Medicines Agency, 2022b). Other sources, such as clinical trials and/or post-marketing studies, were also necessary to identify or confirm safety issues in some alerts. More than one out of every four safety alerts concerned drugs under “additional monitoring,” that is, medicines authorized for use in the EU but are being monitored particularly closely by regulatory authorities either because the medicine has been recently approved or because there are limited data on its long-term use. These findings show that recently marketed drugs, with less safety information available, should be a priority for the reporting of suspected ADRs. Therefore, information about any possible ADRs must be collected as early as possible to further inform healthcare practitioners about the safety of these medicines.

Although most alerts were addressed the adult population, almost half were referred to drugs typically prescribed to elderly patients (whether exclusively or not), which is a population that frequently receives multiple drugs, has multiple morbidities, and is rarely included in clinical trials (Davies and O'Mahony, 2015). Pregnant women and children, who accounted for 12% of the analyzed alerts, are also “vulnerable” populations in terms of drug efficacy/effectiveness and safety. These alerts were specifically addressed to children involved with three drugs (codeine, parenteral nutrition, and minoxidil), which contrasts with another study where antidepressants were the only drug alerts related to children (Clavenna and Bonati, 2009). Spontaneous reporting in the pediatric population is crucial due to the limited number of clinical trials and the huge “off-label” use of drugs in situations where drug safety is not yet well-established (Conroy et al., 2000). Regarding alerts addressed to pregnant women, these mainly concerned the risk of congenital malformations, and the source of information was epidemiological studies (when specified). As pregnant women are traditionally excluded from clinical trials of therapeutics other than those related to diseases of childbirth, these data usually come from observational or pharmacoepidemiological studies (Irl and Hasford, 2020).

Rheumatologists, neurologists (mainly from multiple sclerosis units), and hematologists were most likely interested in the safety alerts. Moreover, almost half of the safety alerts involved drugs restricted to use in the hospital setting. Therefore, hospital pharmacovigilance programs must play an important role in disseminating safety alerts to their healthcare professionals. These results cannot be compared with those of other studies because studies with similar designs are lacking. Findings of this sort are also contingent on the study period assessed and are probably not informative about drug consumption or the number of drugs available for particular medical specialties. ADRs for which alerts are issued are unlikely to be identified in clinical trials because they are rare and unpredictable. In addition, the most frequent types of ADRs in this analysis were infections and neoplasms, which are type A effects related to the mechanism of action of antineoplastic agents, the most frequent drug class alerted, and consequently, these ADRs are frequent and predictable. In contrast to other studies, the drugs most frequently addressed in the alerts reported in this study were antidepressants, antidiabetics, anti-infectives, and anti-inflammatory drugs (Farcaş et al., 2018; Ferreira et al., 2020; Perry et al., 2020; Meddra, 2021). Hepatitis, death, and congenital malformations were the most frequent ADR alerts with the first two being the most frequently reported ADRs in other studies (Farcaş et al., 2018; Meddra, 2021). Nevertheless, information about birth defects is often lacking when a drug is marketed because pregnant women are excluded from clinical trials for ethical reasons. Both reported drugs and ADRs are in line with safety signals assessed by PRAC between 2014 and 2017 (Farcaş et al., 2018).

Regarding the regulatory actions resulting from these alerts, one-third led to a restriction in the use of the medicine, which is a finding that was similar to that reported in another study in which the most frequent restriction involved adding new contraindications for use (Perry et al., 2020). Some of the reported ADRs in this study were sufficiently serious and clinically important enough to require changes to their labeling or to be classified with an unfavorable benefit/risk ratio, leading to their withdrawal from the market. Changes or updates in the SmPC were frequent regulatory actions for almost half of the medicines in this and other studies (Lester et al., 2013; Ferreira et al., 2020; Perry et al., 2020). This was specifically true for nationally authorized drugs, which are more likely to be updated than EU-authorized products (van Hunsel et al., 2021). In contrast, drug withdrawal tended to be infrequent and occurred in approximately 5%–7% of alerted drugs, with several prior informational alerts preceding the suspension of the medicine (Soares et al., 2015; Ferreira et al., 2020). In our study, most of these drugs had been available in the Spanish market for decades. The causes of withdrawal of these drugs were heterogeneous, and for a few drugs the suspension of marketing authorization was due to multiple reasons. The most frequent reported causes for withdrawal from the market were liver, cardiac, and nervous system toxicity, and the median interval between the first report of an ADR pointing to withdrawal and actual withdrawal itself was approximately 1 year (Onakpoya et al., 2016a). However, there are discrepancies in the patterns of withdrawal of medicinal products from the market and withdrawals are inconsistent across countries (Onakpoya et al., 2016b). These findings underline the need for continuous monitoring of risks throughout the lifecycle of a medicine.

### 4.1 Limitations and strengths

This study has several limitations. First, only the alerts issued in Spain were evaluated; however, evaluations and decisions taken by the EMA were involved in more than half of them. A second limitation was the short length of the study period of only 7 years; nonetheless, we consider this period sufficiently representative to characterize the issued alerts. The reason why we did not include alerts issued during 2020 and 2021 should be self-evident because the outbreak of the COVID-19 pandemic quickly changed the profile of alerts and their focus shifted to drugs used to treat the coronavirus and vaccines. Another limitation is that we did not include Direct Healthcare Professional Communications (formerly called ‘Dear Doctor Letter’), which are issued by pharmaceutical companies to inform healthcare providers of important drug-related safety issues and follow a different safety assessment process. Finally, we did not assess the impact of alerts on the prescription of the medicines concerned and trends in their consumption. Nevertheless, the impact of risk minimization has already been assessed in various other studies (The European Network of Centres for Pharmacoepidemiology and Pharmacovigilance).

Our study also has certain strengths. This is the first study to analyze drug alerts issued by the Spanish Medicines Agency. Additionally, it is the first study to devote special attention to alerts involving drugs under “additional monitoring” and also the first to break down the analysis according to the population targeted and the medical specialty involved.

## 5 Conclusions

Pharmacovigilance systems should ensure monitoring of all authorized medicines throughout their lifecycle in clinical use, and regulatory authorities should continuously review their benefit/risk ratio and issue an alert whenever a new signal or risk is detected. Over a 7-year period, almost one hundred alerts were issued by the Spanish’s Medicines Agency. Despite the limitations, spontaneous reporting was the most frequent source of information for the basis of alerts and reinforced the idea that such reporting is an essential tool for drug and patient safety. Drugs “under additional monitoring” were frequently implicated in alerts due to their unknown safety profile. The most frequent regulatory action resulting from an alert was changes in the SmPC, and one in ten alerts resulted in the withdrawal of the drug from the market. Our findings underline the important contribution of the spontaneous reporting of ADRs and the need for a safety assessment throughout the lifecycle of medicines, particularly when ‘vulnerable’ patient populations, such as children and pregnant women, are involved.

## Data Availability

The raw data supporting the conclusions of this article will be made available by the authors without undue reservation.
